# Supplementary material for: Identification of photoperiod-induced specific miRNAs in the adrenal glands of Sunite sheep (Ovis aries)
Source: Front Vet Sci. 2022 Jul 22;9:888207. doi: 10.3389/fvets.2022.888207 (PMC9354845; doi:10.3389/fvets.2022.888207)
Supplement: Supplementary file 1 [file Data_Sheet_1.ZIP › Supplementary file/miRDeep2 file.zip!/miRDeep2 file/Know_Structure/LP42A2/oar-mir-125b.pdf]

miRBase precursor : oar-mir-125b
Total read count : 173658
oar-miR-125b read count : 172893
remaining reads : 765

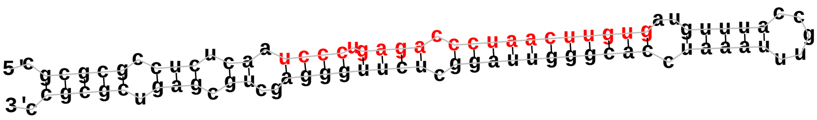

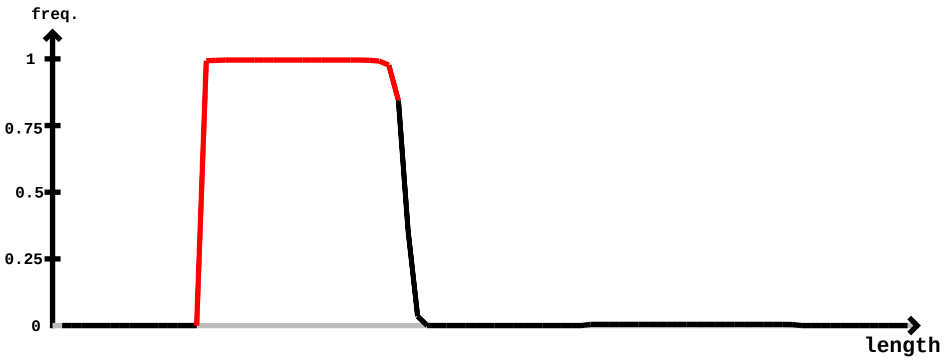


```
                oar-miR-125b
5'- cgcgcgccucucaaucccugagacccuaacuugugauguuuaccguuuaaaauccacggguuaggcucuugggagcugcgagucgcgcc  -3'   exp
    .(((((.(((.((.((((.((((.((((((((((..(((((.....))))).))))))))))).))))))))..)).))).)))))).   reads   mm   sample
    ............aaucccugagacccuaacuugug......................................................   1       0    seq
    .............aucccugagacccuaacuugug......................................................   1       0    seq
    .............aucccugagacccuaacuuguga.....................................................   2       0    seq
    ..............ucccugagacccuaCcu..........................................................   2       1    seq
    ..............ucccugagacccuaacC..........................................................   35      1    seq
    ..............ucccugagacccuaacu..........................................................   154     0    seq
    ..............Acccugagacccuaacu..........................................................   1       1    seq
    ..............ucccuAagacccuaacu..........................................................   1       1    seq
    ..............ucccugagGcccuaacu..........................................................   1       1    seq
    ..............ucccugagacccuaacCu.........................................................   6       1    seq
    ..............ucccugagacccuaacuu.........................................................   426     0    seq
    ..............ucccugagacccuaacuug........................................................   2682    0    seq
    ..............ucccugagacccuaacuugu.......................................................   22967   0    seq
    ..............ucccugagacccuaacuugug......................................................   84207   0    seq
    ..............ucccugagacccuaacuuguga.....................................................   55985   0    seq
    ..............ucccugagacccuaacuugugaG....................................................   1904    1    seq
    ..............ucccugagacccuaacuugugau....................................................   4085    0    seq
    ..............ucccugagacccuaacuugugaug...................................................   18      0    seq
    ..............ucccugagacccuaacuugugaGg...................................................   8       1    seq
    ..............ucccugagacccuaacuugugaugu..................................................   15      0    seq
    ..............ucccugagacccuaacuugugaGgu..................................................   1       1    seq
    ...............cccugagacccuaacuug........................................................   2       0    seq
    ...............cccugagacccuaacuugu.......................................................   7       0    seq
    ...............cccugagacccuaacuugug......................................................   25      0    seq
    ...............cccugagacccuaacuuguga.....................................................   125     0    seq
    ...............cccugagacccuaacuugugaG....................................................   5       1    seq
    ...............cccugagacccuaacuugugau....................................................   3       0    seq
    ................ccugagacccuaacuug........................................................   2       0    seq
    ................ccugagacccuaacuugu.......................................................   38      0    seq
    ................ccugagacccuaacuugug......................................................   116     0    seq
    ................ccugagacccuaacuuguga.....................................................   54      0    seq
    ................ccugagacccuaacuugugaG....................................................   4       1    seq
    ................ccugagacccuaacuugugau....................................................   5       0    seq
    .................cugagacccuaacuugug......................................................   1       0    seq
    .................cugagacccuaacuuguga.....................................................   2       0    seq
```

oar-miR-125b

```
cgcgcgccucucaaucccugagacccuaacuugugauguuuaccguuuaaauccacggguuaggcucuugggagcugcgagucgcgcc
.................cugagacccuaacuugugau...................................................    1    0    seq
...................gagacccuaacuuguga....................................................    1    0    seq
...................................................uccacggguuaggcucuuggga...............    2    0    seq
......................................................acggguuaggcucuuggg................    4    0    seq
......................................................acggguuaggcucuuggga...............    1    0    seq
......................................................acggguuaggcucuugggag..............    5    0    seq
......................................................acggguuaggcucuugggagc.............    72   0    seq
......................................................acggguuaggcucuugggagcu............    672  0    seq
......................................................acggguuaggcucuugggagcug...........    8    0    seq
.......................................................cggguuaggcucuugggagcu............    1    0    seq
```
